# Supplementary material for: Morning exercise and pre‐breakfast metformin interact to reduce glycaemia in people with type 2 diabetes: a randomized crossover trial
Source: J Physiol. 2024 Mar 24;602(23):6491–506. doi: 10.1113/JP285722 (PMC11607888; doi:10.1113/JP285722)
Supplement: Supplementary file 2 — Supporting Information Supplementary Table S1. Baseline characteristics by metformin timing during baseline period. Supplementary Table S2. Baseline characteristics by metformin timing in the morning exercise period. Supplementary Table S3. Baseline characteristics by metformin timing in the evening exercise period. Supplementary Figure S1. Number of exercise sessions completed during trial in the morning and evening exercise periods. Supplementary Figure S2. Time of day of exercise sessions completed during trial in the morning and evening exercise periods Supplementary Figure S3. Total hours of sleep from Garmin devices at baseline and Munich Chronotype Questionnaires Supplementary Figure S4. Sleep architecture in the baseline, and morning and evening exercise periods of the trial. Supplementary Figure S5. Mean area under the curve glucose concentrations over 24 h hourly during baseline, and morning and evening exercise split by sex. Supplementary Figure S6. Mean area under the curve values over 24 h hourly in baseline, and morning and evening exercise periods split by season. Supplementary Figure S7. Meal timing. Supplementary Figure S8. Meal and metformin timing. [file TJP-602-6491-s002.docx]

**SUPPORTING INFORMATION**

**Supplementary table 1**. Baseline characteristics by metformin timing during baseline period. HbA1c, blood glycosylated haemoglobin. Values are mean±SD(n). P values are from unpaired t-test.

| **Baseline period** |  |  |  |
| --- | --- | --- | --- |
| **Characteristic** | **Metformin**  **before**  **breakfast *(n)*** | **Metformin  after  breakfast *(n)*** | ***p* value** |
| Age (years) | 59.1±7.7 (9) | 64.3±8.1 (4) | 0.30 |
| BMI (Kg/m^2^) | 30.6±3.8 (9) | 30.8±2.4 (5) | 0.91 |
| HbA1c (mmol/mol) | 59.6±13.9 (9) | 68.8±14.9 (5) | 0.26 |
| HbA1c (%) | 7.6±1.3 (9) | 8.4±1.4 (5) | 0.27 |
| Time since T2D diagnosed (years) | 8.4±7.7 (9) | 10.7±1.5 (3) | 0.64 |
| Dose of Metformin per day (mg) | 1918.0±1496.0 (8) | 1333.0±577.4 (3) | 0.52 |
| Time of Metformin (years) | 6.4±3.3 (8) | 3.7±4.6 (3) | 0.29 |
| Number of steps | 7396±3074 (9) | 6133±1792 (5) | 0.42 |

**Supplementary table 2**. Baseline characteristics by metformin timing in the morning exercise period. HbA1c, blood glycosylated haemoglobin. Values are mean±SD(n). P values are from unpaired t-test.

| **Morning exercise** |  |  |  |
| --- | --- | --- | --- |
| **Characteristic** | **Metformin before breakfast *(n)*** | **Metformin  after  breakfast *(n)*** | ***p* value** |
| Age (years) | 61.75±7.6 (8) | 63.4±7.8 (5) | 0.71 |
| BMI (Kg/m^2^) | 30.8±4.0 (8) | 30.8±2.4 (5) | 0.99 |
| HbA1c (mmol/mol) | **56.9±12.1 (8)** | **75.2±10.5 (5)** | **0.01** |
| HbA1c (%) | **7.3±1.2 (6)** | **9.0±1.0 (5)** | **0.02** |
| Time since T2D diagnosed (years) | **6.3±4.2 (8)** | **16.3±8.4 (3)** | **0.02** |
| Dose of Metformin per day (mg) | 1357.0±475.6(7) | 1167±288.7(3) | 0.54 |
| Time of Metformin (years) | 5.6±2.6 (7) | 4.7±6.4 (3) | 0.74 |
| Number of steps | 7056±3099 (8) | 6752±2551 (5) | 0.86 |

**Supplementary table 3**. Baseline characteristics by metformin timing in the evening exercise period. HbA1c, blood glycosylated haemoglobin. Values are mean±SD(n). P values are from unpaired t-test.

| **Evening exercise** |  |  |  |
| --- | --- | --- | --- |
| **Characteristic** | **Metformin before breakfast *(n)*** | **Metformin  after  breakfast *(n)*** | ***p* value** |
| Age (years) | 60.9±5.2 (8) | 61.0±15.0 (3) | 0.98 |
| BMI (Kg/m^2^) | 30.5±3.9 (8) | 32.6±2.5 (3) | 0.41 |
| HbA1c (mmol/mol) | 66.7±16.2 (8) | 60.3±13.2 (3) | 0.55 |
| HbA1c (%) | 8.2±1.48 (8) | 7.7±1.7 (3) | 0.55 |
| Time since T2D diagnosed (years) | 11.0±8.3 (6) | 9.0±4.4 (3) | 0.71 |
| Dose of Metformin per day (mg) | 1333.0±408.2 (6) | 1333.0±577.4 (3) | 0.99 |
| Time of Metformin (years) | **8.2±2.4 (5)** | **2.0±1.7 (3)** | **0.008** |
| Number of steps | 6264±2145 (8) | 5603±1938 (3) | 0.65 |


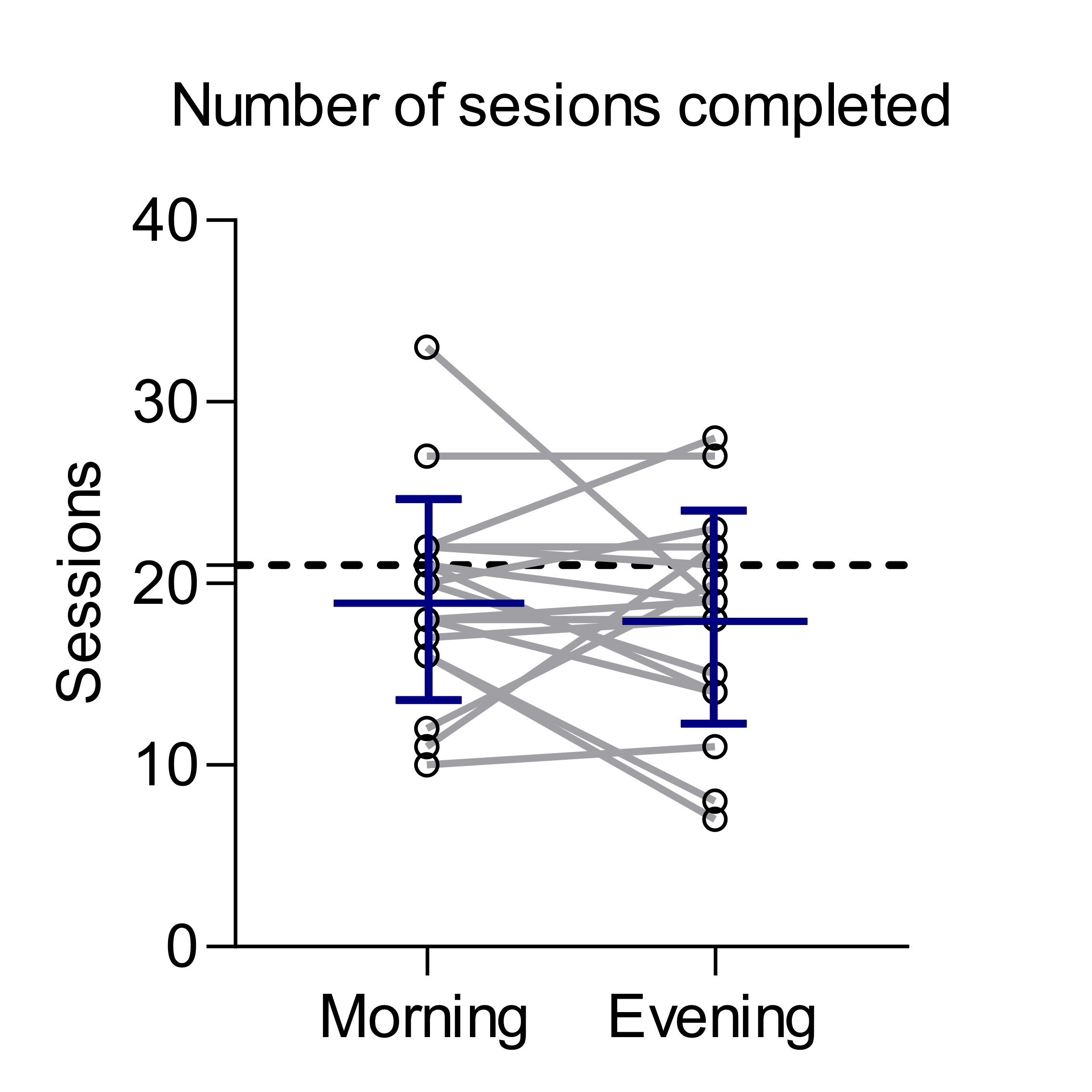


**Supplementary Figure 1.** Number of exercise sessions completed during trial in the morning and evening exercise periods (n=18), blue lines are mean±SD, the dotted line represents the number of sessions participants were asked to complete. Data were analysed using paired t-test.


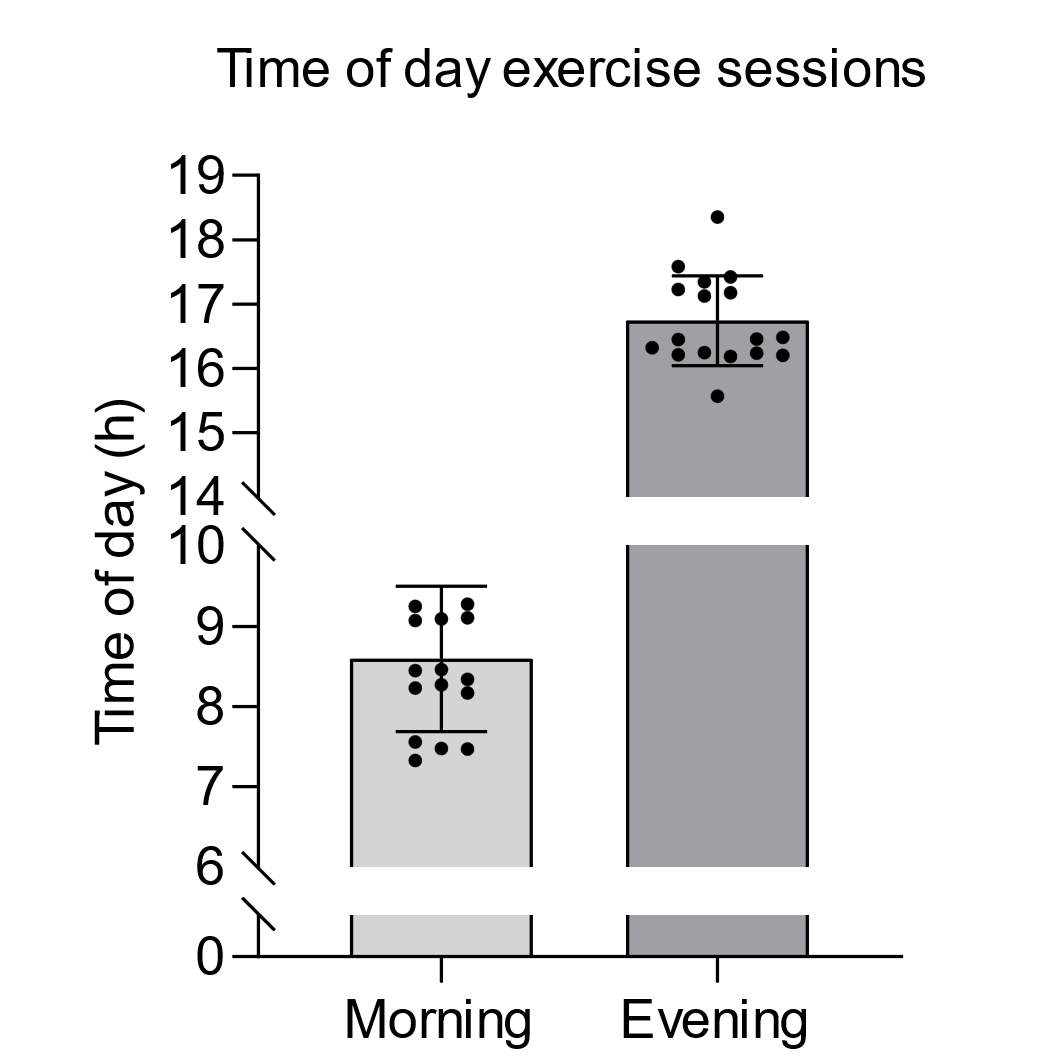


**Supplementary Figure 2.** Time of day of exercise sessions completed during trial in the morning and evening exercise periods (n=18). Values are mean±SD.


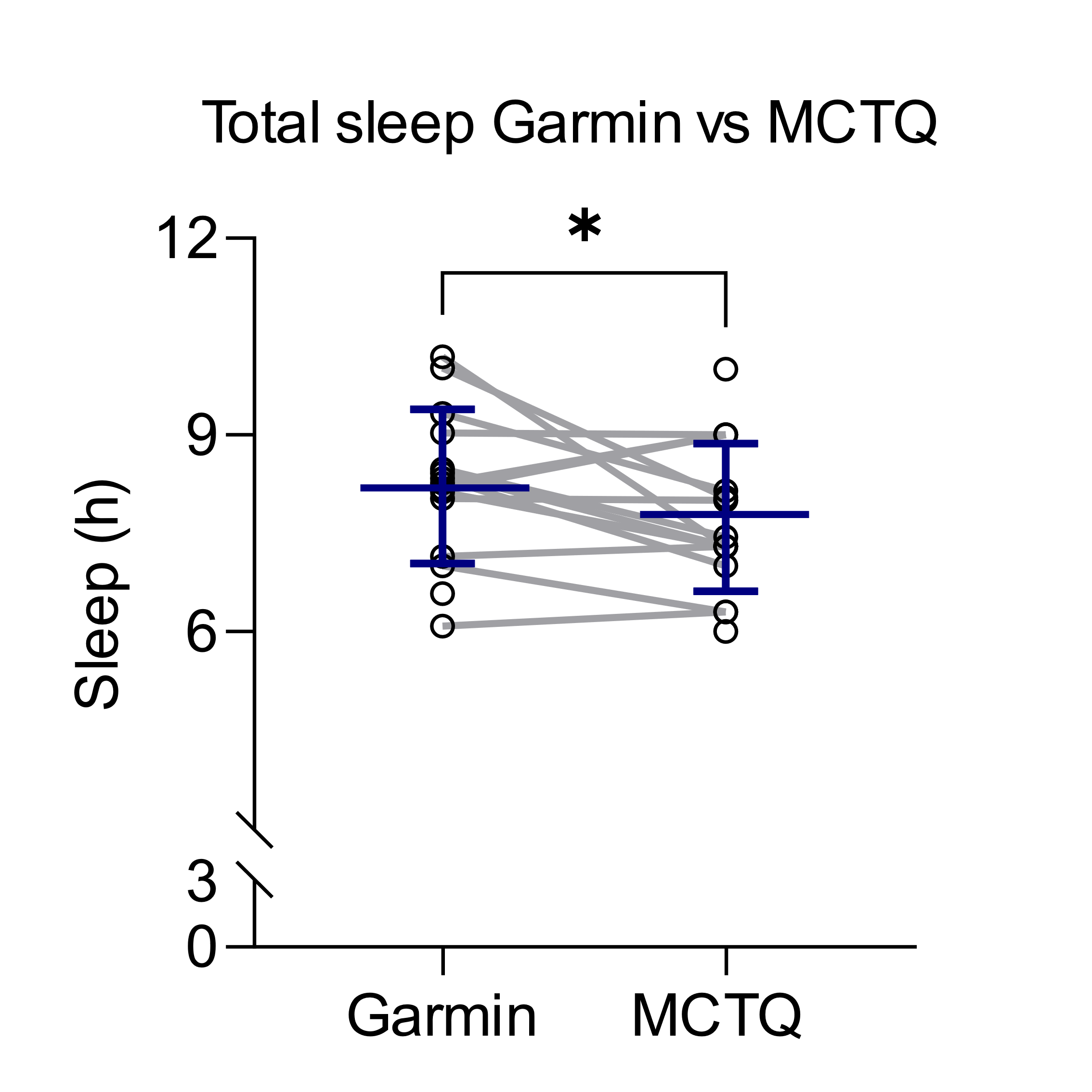


**Supplementary Figure 3.** Total hours of sleep from Garmin devices at baseline (Garmin) and Munich Chronotype Questionnaires (MCTQ) (n=14), blue lines are mean±SD, data were analysed using paired t-test. *=p<0.05.


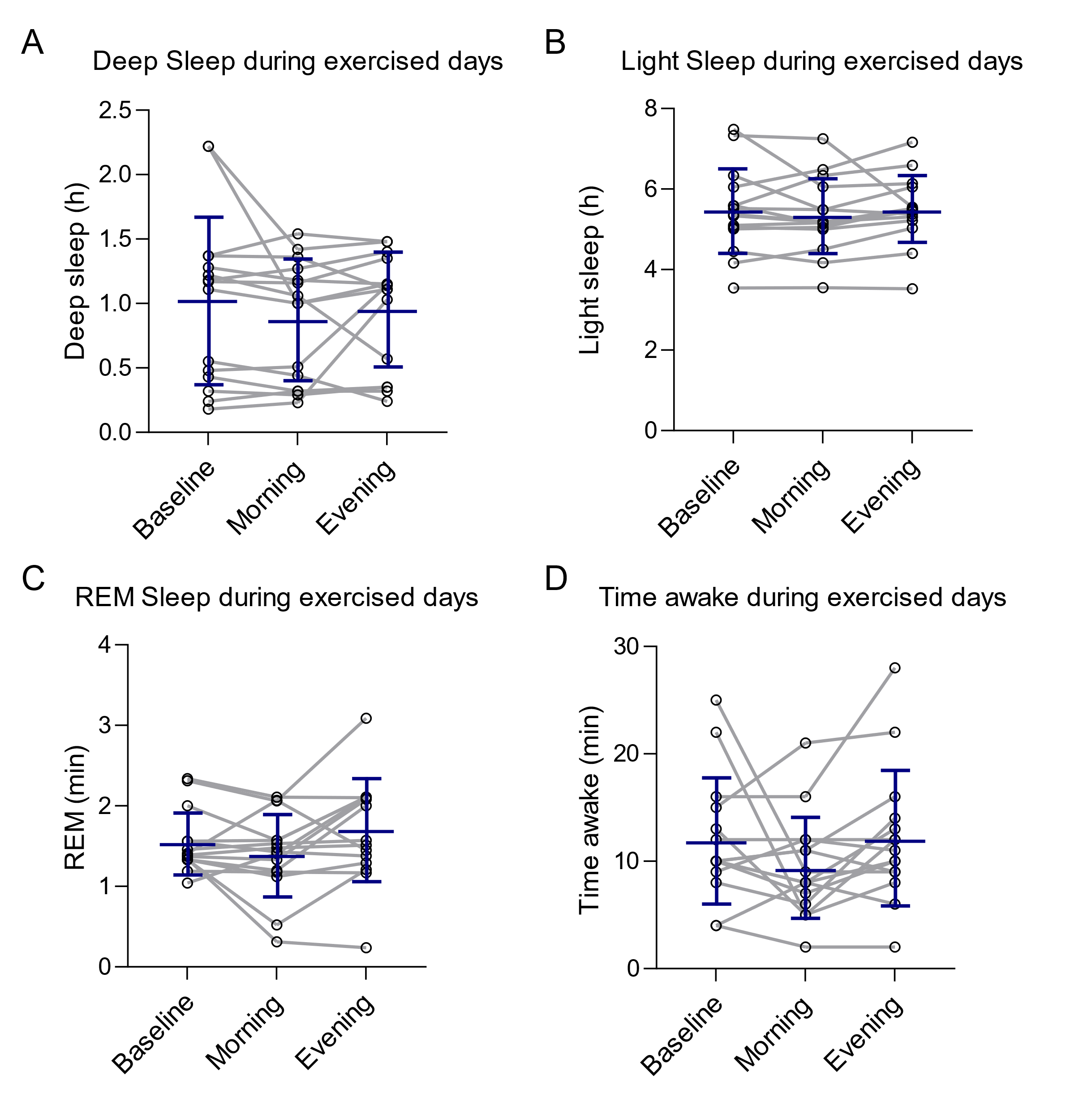


**Supplementary Figure 4**. Sleep architecture in the baseline, and morning and evening exercise periods of the trial. The values are shown (A) Deep sleep (n=15), (B) Light sleep (n=15), (C) REM sleep (n=15) and (D) Time awake (n=15), blue lines are mean±SD. Data were analysed using one-way ANOVA followed up by Holm-Šídák's multiple comparisons test.

**
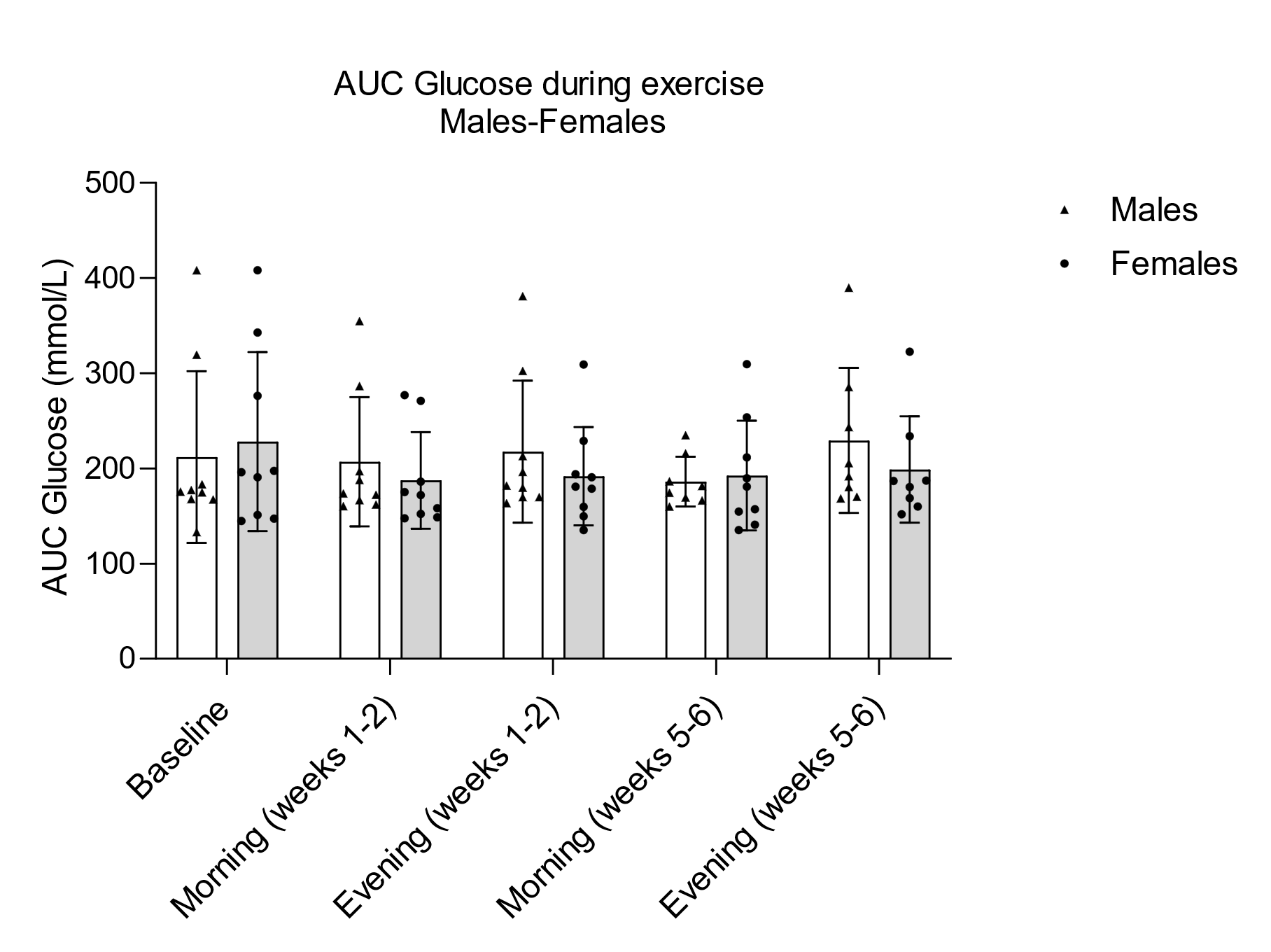
**

**Supplementary figure 5**. Mean area under the curve (AUC) glucose concentrations over 24-hours hourly during baseline, and morning and evening exercise split by sex. Glucose levels in Males weeks 1-2 (n=9), weeks 5-6 (n=8); Females weeks 1-2 (n=9), weeks 5-6 (n=8). Values are mean±SD. Data were analysed using two-way mixed-model ANOVA followed up by Holm-Šídák's multiple comparisons test.


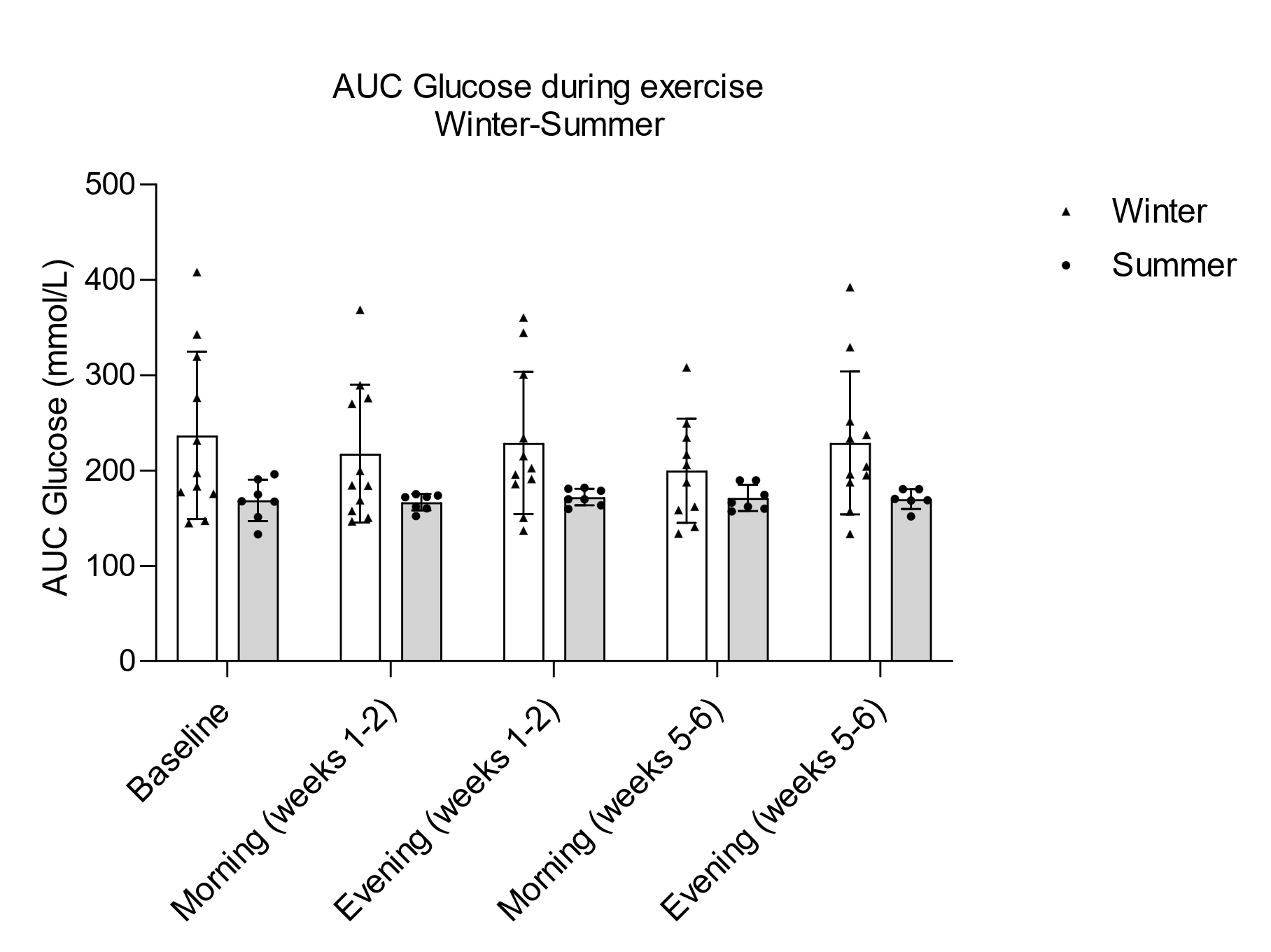


**Supplementary Figure 6**. Mean area under the curve (AUC) values over 24-hours hourly in baseline, and morning and evening exercise periods split by season. Winter weeks 1-2 (n=11), weeks 5-6 (n=10). Summer weeks 1-2 (n=7), weeks 5-6 (n=6). Values are mean±SD. Data were analysed using two-way mixed-model ANOVA followed up by Holm-Šídák's multiple comparisons test.


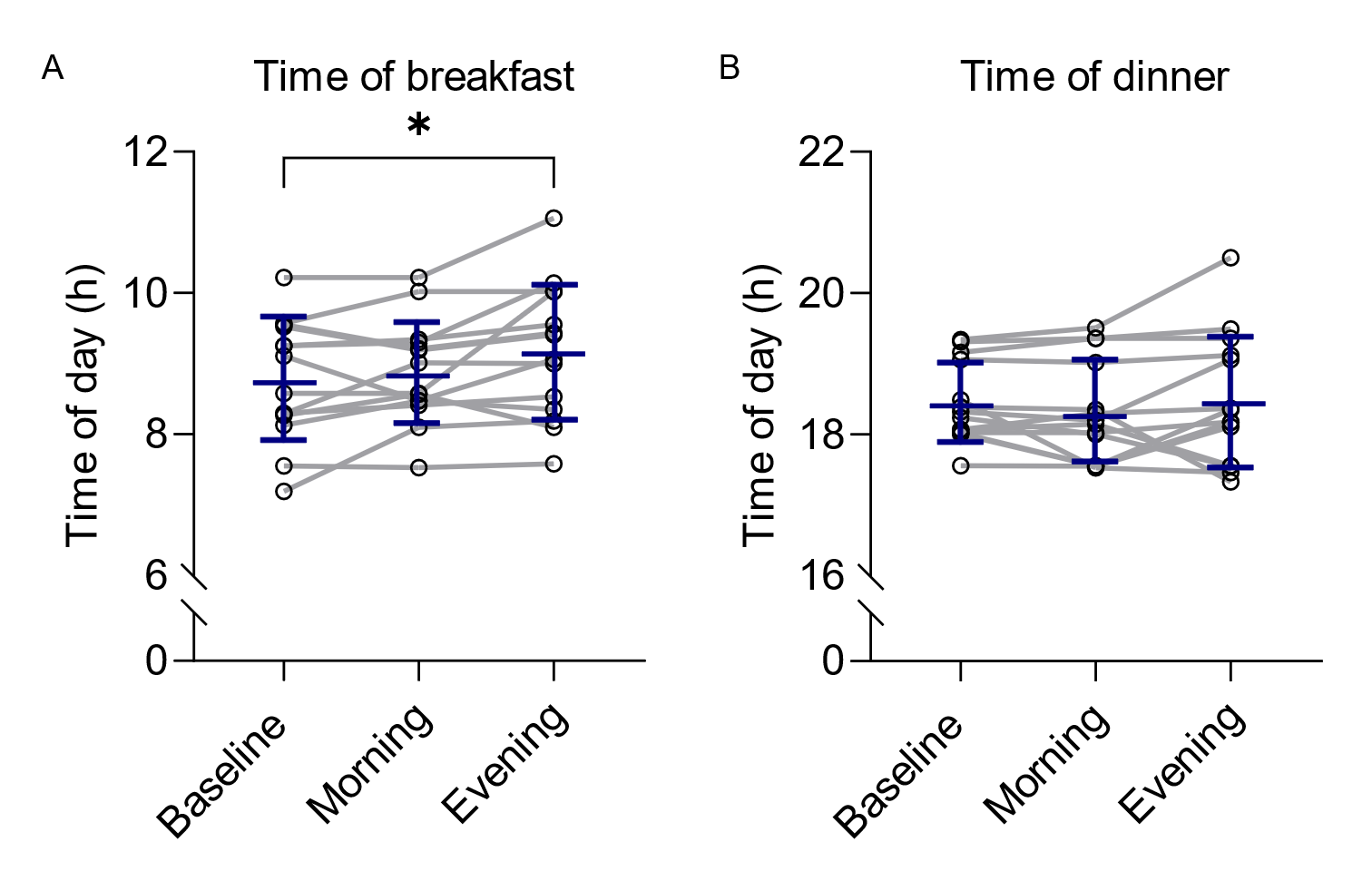


**Supplementary Figure 7.** Meal timing. Data collected from self-reported 4-days food diaries for (A) Time of brekfast (n=14), (B) Time of dinner (n=14). Blue lines are mean±SD. Data were analysed using one-way ANOVA followed up by Holm-Šídák's multiple comparisons test.


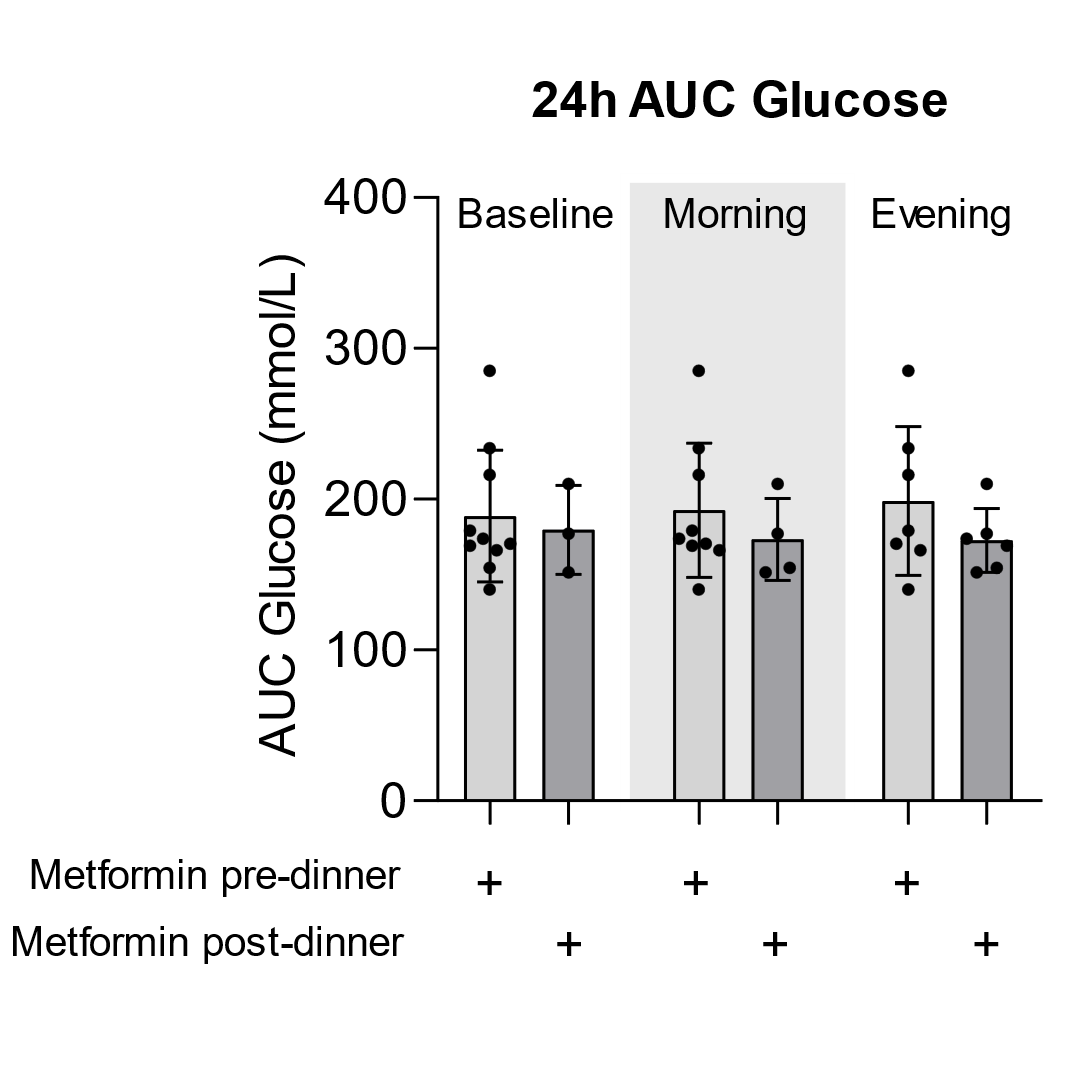


**Supplementary Figure 8.** Meal and metformin timing. Mean area under the curve (AUC) glucose values for the first 24 hours of exercise day comparing Metformin pre and post dinner. (A) Baseline, metformin pre-dinner (n=10), metformin post-dinner (n=3); (B) Morning exercise, metformin pre-dinner (n=9), metformin post-dinner (n=4); (C) Evening exercise, metformin pre-dinner (n=7), metformin post-dinner (n=6). Values are mean±SD. Data were analysed using unpaired t-test.
